# Multi-trait GWAS identifies pleiotropic loci shared between early pregnancy bleeding and psychiatric traits

**DOI:** 10.3389/fpsyt.2026.1911755

**Published:** 2026-08-27

**Authors:** Deyu Ji, Xianjin Wang, Meng Zhao, Chunchen Liu, Kai Sun

**Affiliations:** 1 First Clinical Medical College, Nanchang University, Nanchang, Jiangxi, China; 2 College of Medical Information and Artificial Intelligence, Shandong First Medical University, Jinan, Shandong, China; 3 Shandong Engineering Research Center of Intelligent Surgery, The First Affiliated Hospital of Shandong First Medical University, Jinan, China; 4 Shandong Data Open Innovative Application Laboratory, The First Affiliated Hospital of Shandong First Medical University, Jinan, China; 5 Shandong Key Laboratory of Digital Diagnosis and Treatment of Thoracic Oncology, The First Affiliated Hospital of Shandong First Medical University, Jinan, China; 6 China Foundation for Youth Entrepreneurship and Employment-Incaier Public Welfare Fund, Beijing, China

**Keywords:** early pregnancy bleeding, genetic correlation, local genetic correlation, multi-trait genome-wide association study, neuroendocrine, pleiotropy, psychiatric disorders, RMDN3

## Abstract

**Introduction:**

Early pregnancy bleeding is a common pregnancy complication, yet its genetic basis and potential links with psychiatric traits remain poorly understood. This study aimed to characterize the shared genetic architecture between early pregnancy bleeding and reproductive, psychiatric, and cardiometabolic traits.

**Methods:**

We integrated linkage disequilibrium score regression (LDSC), local genetic correlation analysis (LAVA), and multi-trait genome-wide association analysis (MTAG). LDSC was used to estimate genome-wide genetic correlations, including sex-stratified analyses. LAVA was applied to identify genomic regions contributing to local genetic sharing. Guided by these correlation patterns, MTAG was performed to improve locus discovery, followed by cis-eQTL analysis using GTEx v8 to explore potential regulatory mechanisms.

**Results:**

LDSC revealed significant positive genetic correlations between early pregnancy bleeding and reproductive traits, including endometriosis, miscarriage, and uterine fibroids. Strong positive correlations were also observed with several psychiatric disorders, including major depressive disorder, post-traumatic stress disorder, and attention deficit hyperactivity disorder. Sex-stratified analyses suggested stronger genetic correlations with emotional reactivity-related traits in females, whereas social and behavioral traits were more prominent in males. LAVA localized these shared signals to specific genomic regions and identified pleiotropic hotspots at 8q21 near *RUNX1T1* and 9p21 near *CDKN2A/B*. MTAG identified two novel loci, 15q15.1 marked by rs45457497 and 11q13.1 marked by rs2452681. Cis-eQTL analysis showed that the lead variant at 15q15.1 regulates *RMDN3* expression across multiple brain regions, while the 11q13.1 locus regulates *PACS1, GAL3ST3*, and *SF3B2* expression in brain tissues and the pituitary.

**Discussion:**

These findings position early pregnancy bleeding as a multifactorial trait shaped by shared reproductive, psychiatric, neuroendocrine, and stress-related biology. The implication of *RMDN3*, which encodes a mitochondrial outer membrane protein involved in ER-mitochondria tethering and calcium homeostasis, suggests a potential molecular link between neuroendocrine stress pathways, psychiatric susceptibility, and reproductive vulnerability.

## 1 Introduction

Pregnancy-associated bleeding can occur at all stages of pregnancy, ranging from early gestational bleeding to antepartum hemorrhage and postpartum hemorrhage (PPH). These bleeding complications are major causes of maternal morbidity and mortality worldwide and impose a substantial burden on maternal and fetal health (1). Despite their clinical importance, the genetic basis of pregnancy-associated bleeding has remained poorly understood.

Recent advances in genome-wide association studies (GWAS) have begun to shed light on the inherited component of pregnancy-related bleeding. In particular, a large-scale meta-analysis identified five genome-wide significant loci associated with PPH, implicating genes involved in uterine contractility and progesterone signaling (2). In contrast, bleeding in early pregnancy yielded no genome-wide significant signals, although it exhibited strong polygenic architecture and notable genetic correlations with a range of human traits, including anthropometric measures, reproductive traits, and, intriguingly, psychiatric disorders. These findings suggest that while early pregnancy bleeding may not be driven by single loci of large effect, it may share a complex genetic background with diverse physiological and neuropsychiatric traits.

To uncover such shared genetic influences, cross-trait analysis methods have been developed, which leverage genetic correlations across multiple phenotypes to improve statistical power and refine association signals. These approaches integrate summary-level GWAS data from different traits, enabling the detection of loci that are consistently associated with related phenotypes but may not reach genome-wide significance in individual studies (3). By combining information across traits, these methods facilitate the identification of pleiotropic loci and enhance our understanding of the common genetic architecture underlying clinically overlapping conditions (4–6).

In this study, we aimed to characterize the shared genetic architecture between early pregnancy bleeding and reproductive, psychiatric, and cardiometabolic traits through an integrative genetic framework. We first applied linkage disequilibrium score regression (LDSC) to identify genome-wide genetic correlations between early pregnancy bleeding and related traits, followed by local genetic correlation analysis (LAVA) to determine whether these shared signals were concentrated in specific genomic regions. Based on the observed cross-trait genetic overlap, we then performed multi-trait genome-wide association analysis (MTAG) to enhance locus discovery by leveraging correlated traits. Finally, we integrated tissue-level and single-cell eQTL annotations to prioritize candidate genes and provide functional insights into the identified loci. Through this stepwise approach, we sought to elucidate the genetic links connecting reproductive vulnerability with psychiatric and neuroendocrine-related biology.

## 2 Results

### 2.1 Genetic correlation between early pregnancy bleeding and reproductive traits

**Figure 1:**
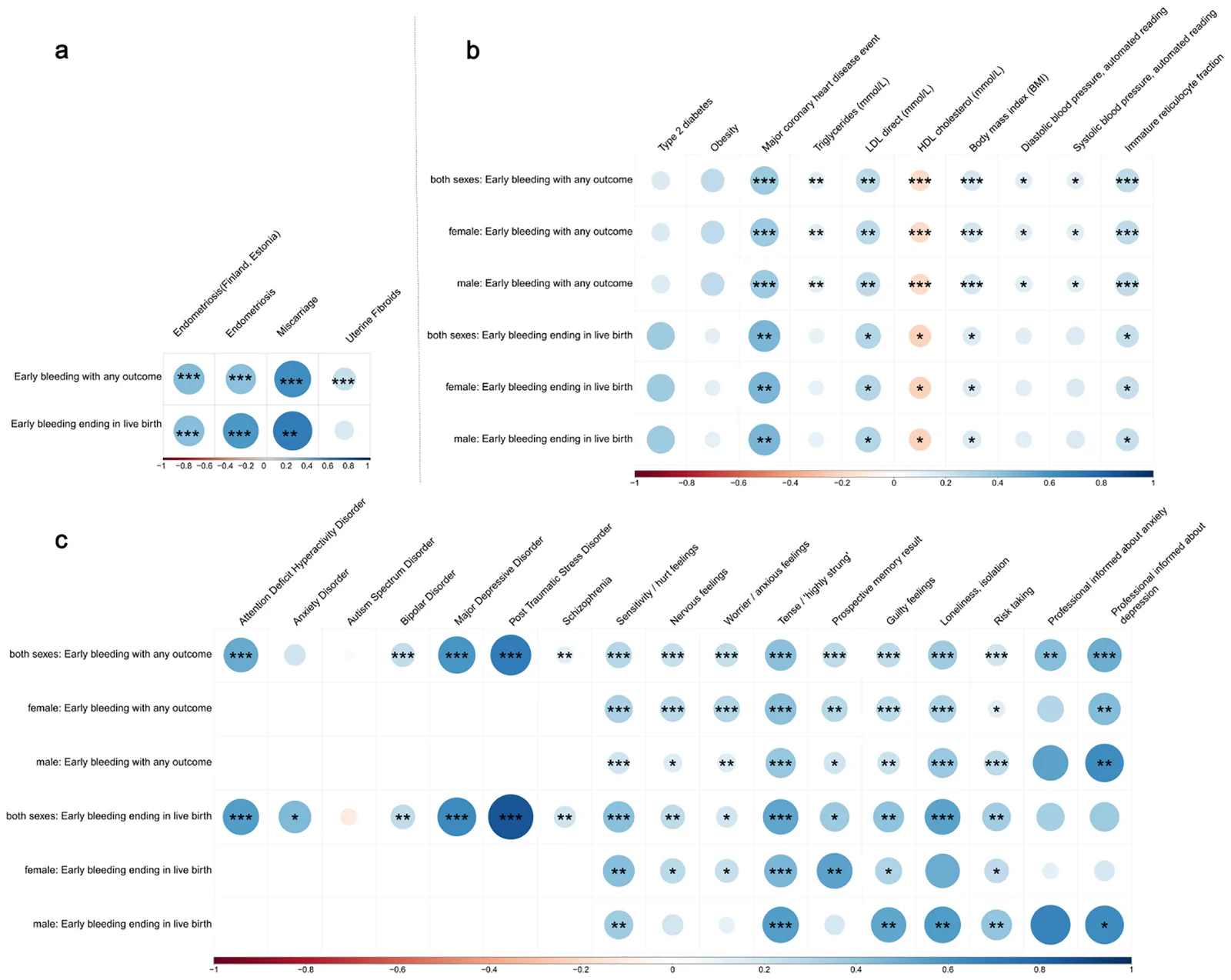
Genome-wide genetic correlations between early pregnancy bleeding and complex traits estimated by LDSC. **(A)** Genetic correlations with gynecological traits including endometriosis, miscarriage, and uterine fibroids. **(B)** Genetic correlations with cardiometabolic and anthropometric traits, stratified by sex. **(C)** Genetic correlations with psychiatric disorders and mental health–related behavioral traits from the PGC and UK Biobank, stratified by sex. Circle size and color intensity represent the magnitude of genetic correlation (rg), with blue indicating positive and red indicating negative correlations. Asterisks denote statistical significance (*P < 0.05, **P < 0.01, ***P < 0.001).

We assessed genome-wide genetic correlations between early pregnancy bleeding and a series of reproductive disorders using linkage disequilibrium score regression. Both bleeding phenotypes—across all pregnancy outcomes (EBA) and restricted to live births (EBL)—showed consistent positive correlations with endometriosis, based on two large European-ancestry GWAS datasets: a pan-European meta-analysis (EBA: r_g_ = 0.41, *p* = 1.0×10^-8^; EBL: r_g_ = 0.58, *p* = 6.0×10^-4^) and a Northern European cohort (EBA: r_g_ = 0.43, *p* = 4.7×10^-8^; EBL: r_g_ = 0.43, *p* = 6.0×10^-4^). Early pregnancy bleeding also exhibited strong genetic overlap with miscarriage (EBA: r_g_ = 0.61, *p* = 9.5×10^-5^; EBL: r_g_ = 0.70, *p* = 0.009) and a moderate correlation with uterine fibroids (EBA: r_g_ = 0.24, *p* = 2.0×10^-5^; EBL: r_g_ = 0.17, *p* = 0.11) (**Supplementary Table 2**; **Figure 1A**). The significant genetic correlations with endometriosis and miscarriage remained significant after false discovery rate (FDR) correction (q < 0.05).

### 2.2 Genetic correlation between early pregnancy bleeding and psychiatric traits

We next evaluated the genetic correlations between early pregnancy bleeding and major psychiatric phenotypes using large-scale GWAS summary statistics from the Psychiatric Genomics Consortium (PGC). Both bleeding phenotypes—across EBA and EBL—showed significant positive correlations with major depressive disorder (EBA: r_g_ = 0.59, *p* = 3.5×10^-28^; EBL: r_g_ = 0.63, *p* = 2.9×10^-7^), post-traumatic stress disorder (EBA: r_g_ = 0.70, *p* = 4.9×10^-25^; EBL: r_g_ = 0.86, *p* = 2.0×10^-6^), and attention deficit hyperactivity disorder (EBA: r_g_ = 0.50, *p* = 3.1×10^-18^; EBL: r_g_ = 0.55, *p* = 1.0×10^-5^). All major psychiatric correlations highlighted above remained significant after FDR correction (q < 0.05). Moderate positive correlations were also observed with bipolar disorder (EBA: r_g_ = 0.24, *p* = 5.8×10^-6^; EBL: r_g_ = 0.25, *p* = 5.2×10^-3^) (**Supplementary Table 3**; **Figure 1C**).

To further examine the relationship between early pregnancy bleeding and mental health–related phenotypes, we extended the analysis to the UK Biobank using the Neale Lab GWAS round 2 resource (http://www.nealelab.is/uk-biobank), which provides sex-stratified summary statistics for behavioral and psychological traits. Several affective and cognitive phenotypes showed notable genetic correlations with early pregnancy bleeding, particularly sensitivity or hurt feelings, nervous feelings, worrier or anxious feelings, tense or “highly strung” personality, prospective memory performance, and guilty feelings. These traits exhibited stronger correlations in females, suggesting that emotional reactivity and stress responsiveness may play a more prominent genetic role in women’s susceptibility to early bleeding events. In contrast, traits reflecting social and behavioral domains—including loneliness and isolation, risk-taking tendency, and self-reported psychiatric diagnoses such as professional consultation for anxiety or depression—showed stronger genetic correlations in males (**Supplementary Table 3**).

### 2.3 Genetic correlation between early pregnancy bleeding and anthropometric traits

Extending beyond reproductive and psychiatric domains, we investigated metabolic and cardiovascular traits from the UK Biobank (Neale Lab v2) in relation to both early bleeding phenotypes (EBA and EBL). Both phenotypes showed nominally positive genetic correlations with several metabolic and cardiovascular traits, including type 2 diabetes, obesity, and major coronary heart disease events. Among these traits, major coronary heart disease events showed robust FDR-significant correlations with early pregnancy bleeding across both phenotypes and sexes, whereas type 2 diabetes and obesity showed weaker or sex-specific associations. Consistent with these findings, triglycerides also demonstrated positive and female-strong correlations, supporting a shared metabolic pathway involving lipid and glucose regulation in women. In contrast, LDL cholesterol exhibited positive correlations that were more pronounced in males, indicating sex-specific lipid mechanisms underlying cardiovascular and reproductive physiology. (**Supplementary Table 4**; **Figure 1B**)

For other anthropometric measures, BMI was positively correlated with early pregnancy bleeding in both EBA and EBL; the effect was similar between sexes in EBA but slightly stronger in females within EBL. Blood pressure traits, including both systolic and diastolic measures, showed broadly positive correlations that were comparable across sexes, suggesting largely shared vascular regulatory influences. (**Supplementary Table 4**; **Figure 1B**)

### 2.4 Local genetic correlation analysis

**Figure 2:**
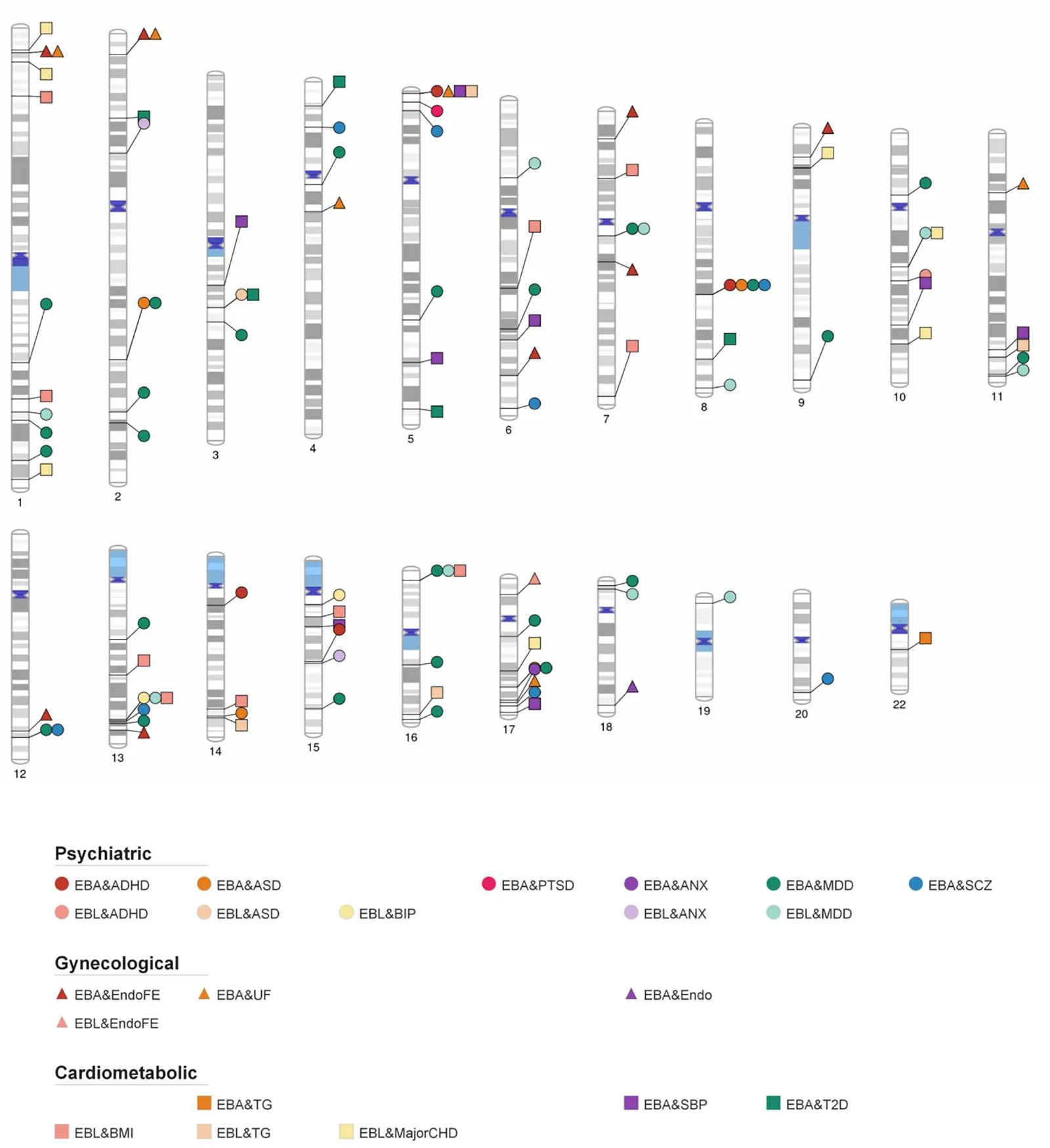
Significant local genetic correlations between early pregnancy bleeding and complex traits identified by LAVA. Chromosomal ideogram showing loci with significant local genetic correlations between early pregnancy bleeding phenotypes (EBA and EBL) and psychiatric (circles), gynecological (triangles), and cardiometabolic (squares) traits. Each symbol is color-coded by trait pair as shown in the legend.

To localize the genome-wide correlations observed in LDSC, we applied LAVA to estimate local genetic correlations across approximately 2,000 independent genomic regions. Several loci exhibited significant local correlations between early pregnancy bleeding and reproductive traits (**Supplementary Table 5**; **Figure 2**). Notably, a strong positive correlation was identified on chromosome 2 (11.2–12.0 Mb), encompassing *GREB1*, a gene previously reported to be associated with endometriosis (7). This region also includes *ROCK2* and *LPIN1*, suggesting a shared regulatory architecture related to hormonal signaling and uterine tissue remodeling.

In addition to reproductive traits, local genetic correlation analysis revealed several regions shared between early pregnancy bleeding and major psychiatric disorders (**Supplementary Table 6**; **Figure 2**). A prominent signal on chromosome 8 (92.9–95.0 Mb) showed strong positive local correlations with ADHD, ASD, schizophrenia, and bipolar disorder. This region contains genes previously implicated in neuronal development and synaptic regulation including *RUNX1T1*. The convergent correlations across multiple psychiatric phenotypes suggest a pleiotropic effect of this locus, potentially linking neurodevelopmental and stress-response pathways to the susceptibility of early pregnancy bleeding.

Beyond reproductive and psychiatric domains, additional loci showing local correlations with metabolic and cardiovascular traits were identified (**Supplementary Table 7**; **Figure 2**). Notably, a strong negative local correlation was observed at the chr9p21 (*CDKN2A/B/ANRIL*) locus, a well-established region involved in vascular remodeling and coronary artery disease (8). This finding suggests that genetic variation at 9p21 may contribute to early pregnancy bleeding through shared mechanisms regulating vascular integrity and endothelial function.

### 2.5 Multi-trait GWAS analysis between early pregnancy bleeding and psychiatric traits

**Figure 3:**
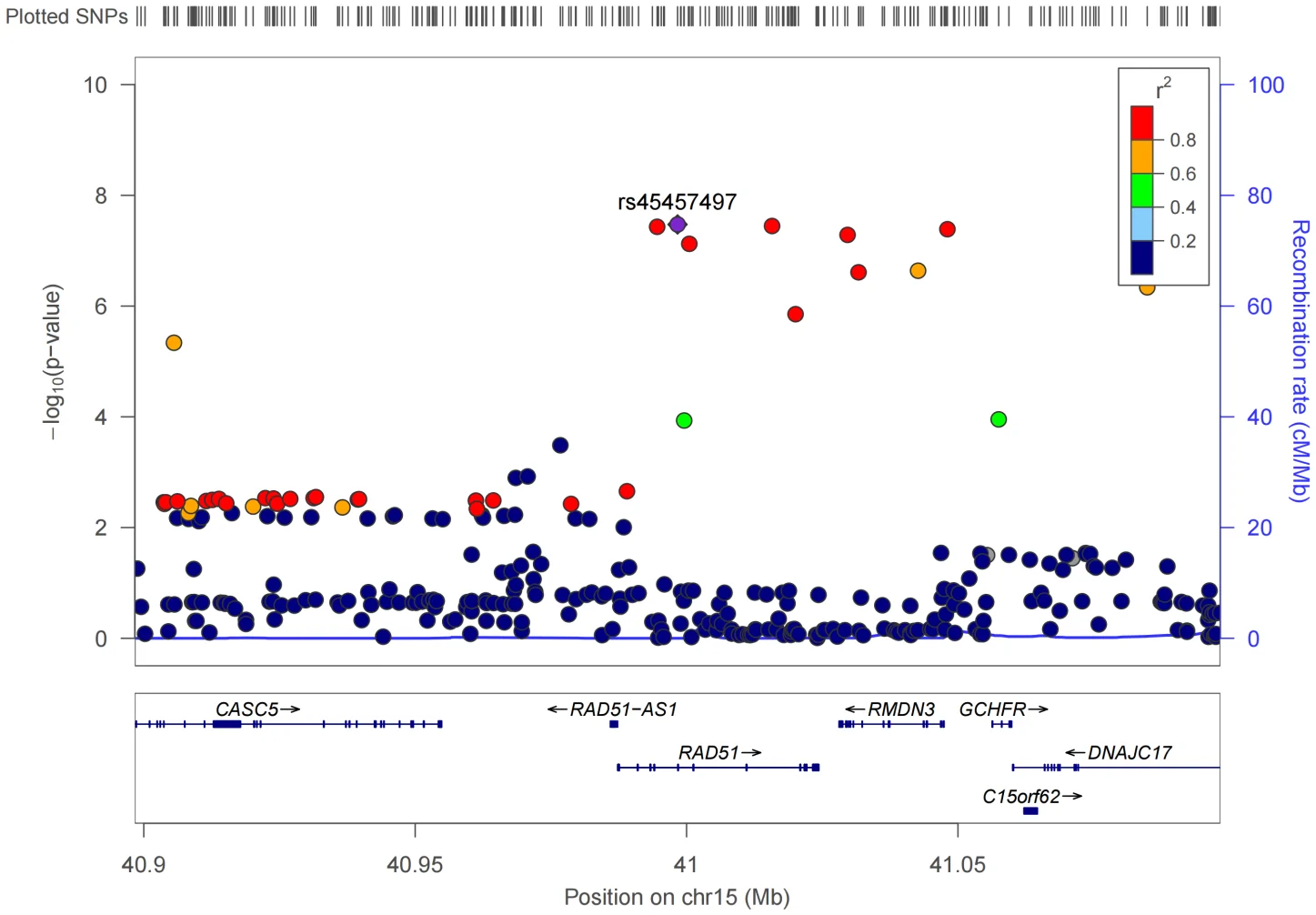
Regional association plot of the novel locus at 15q15.1 identified by MTAG. LocusZoom plot showing the association signals at the 15q15.1 locus (lead SNP rs45457497) from the multi-trait analysis of early pregnancy bleeding and ADHD. SNPs are colored by linkage disequilibrium (r^2^) with the lead variant. Gene annotations and recombination rates (blue line) are based on the hg19 reference.

**Figure 4:**
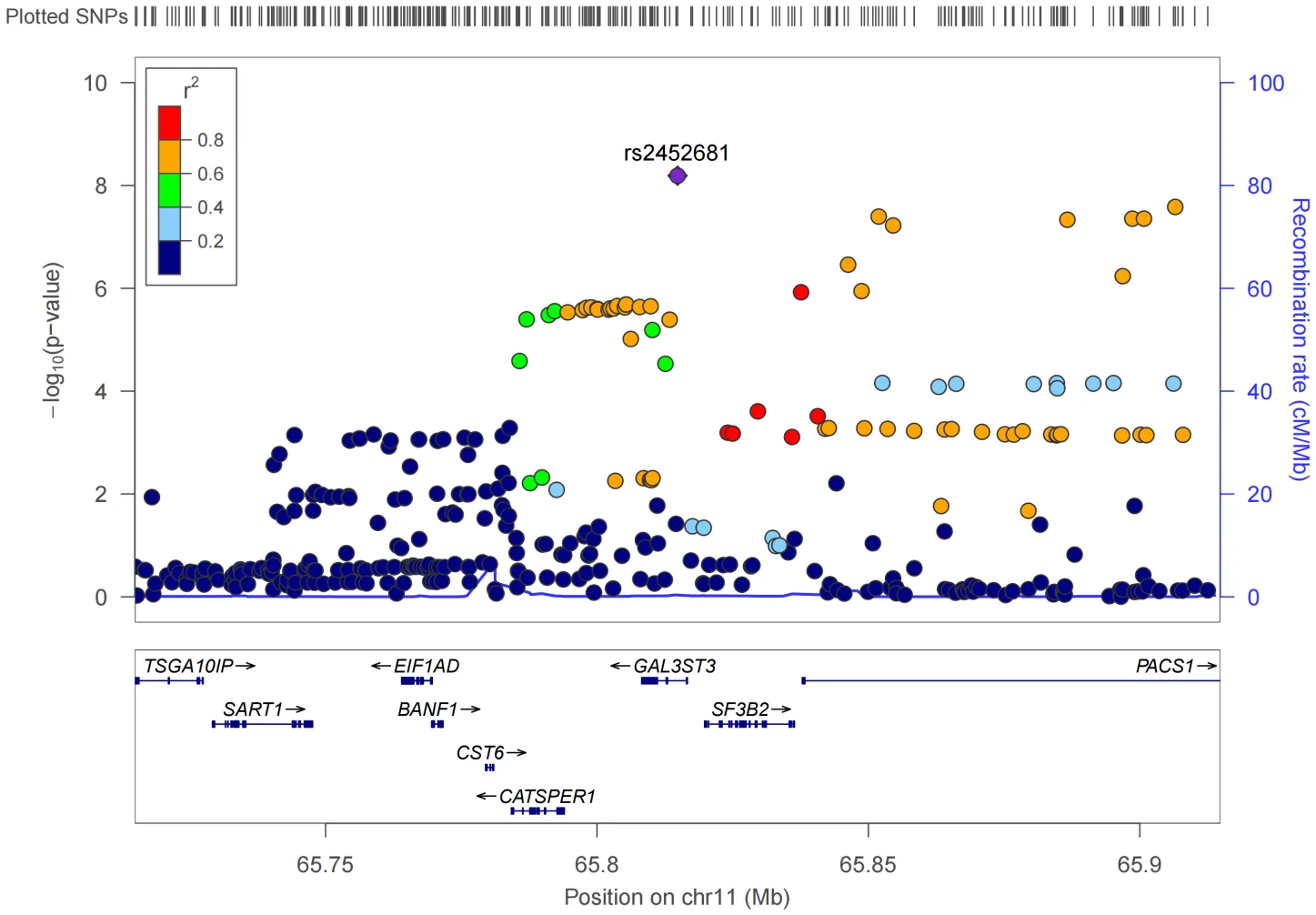
Regional association plot of the novel locus at 11q13.1 identified by MTAG. LocusZoom plot showing the association signals at the 11q13.1 locus (lead SNP rs2452681) from the multi-trait analysis of early pregnancy bleeding and bipolar disorder. SNPs are colored by r^2^ with the lead variant. Gene annotations and recombination rates are based on hg19.

Given the strong genome-wide and local correlations with psychiatric phenotypes, we further conducted MTAG combining early pregnancy bleeding with major psychiatric disorders (**Supplementary Table 8**). Several loci reached genome-wide significance, including previously known regions at 6p21.32 and 12q24.11, consistent with shared immune and calcium signaling pathways. Importantly, two novel associations emerged at 15q15.1 (rs45457497)(**Figure 3**) and 11q13.1 (rs2452681)(**Figure 4**). Neither locus has been previously reported in relation to psychiatric traits or pregnancy-related outcomes, suggesting potential new biological connections between neurodevelopmental regulation and reproductive physiology.

**Figure 5:**
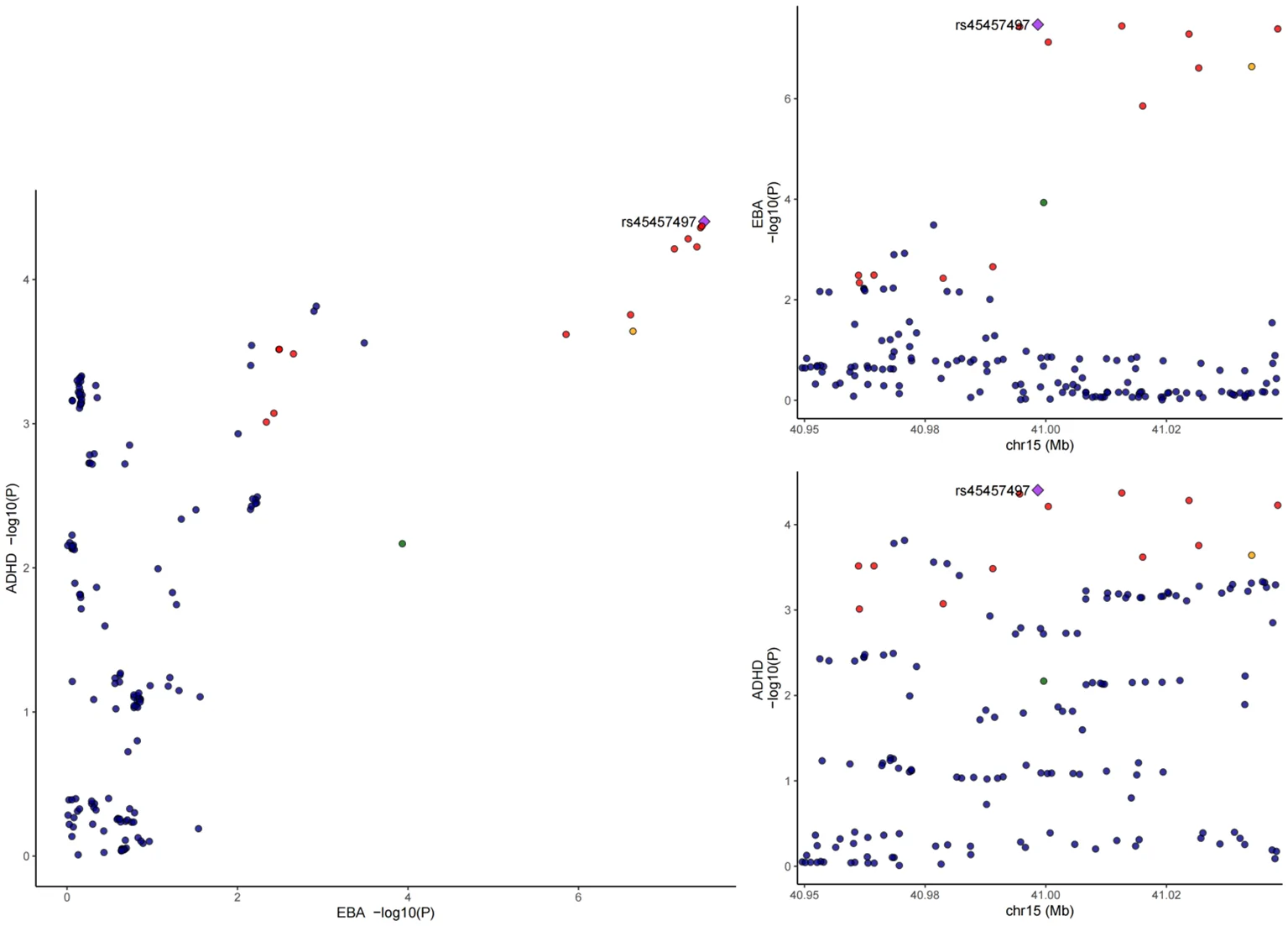
LocusCompare plot of the 15q15.1 locus between early pregnancy bleeding and ADHD. Comparison of association signals at the 15q15.1 locus (lead SNP rs45457497) between EBA and ADHD. The left panel shows a scatter plot of −log_10_(*P*) values for each SNP in both traits. The right panels show the regional association signals for EBA (top) and ADHD (bottom) separately. SNPs are colored by r^2^ with the lead variant (purple diamond).

**Figure 6:**
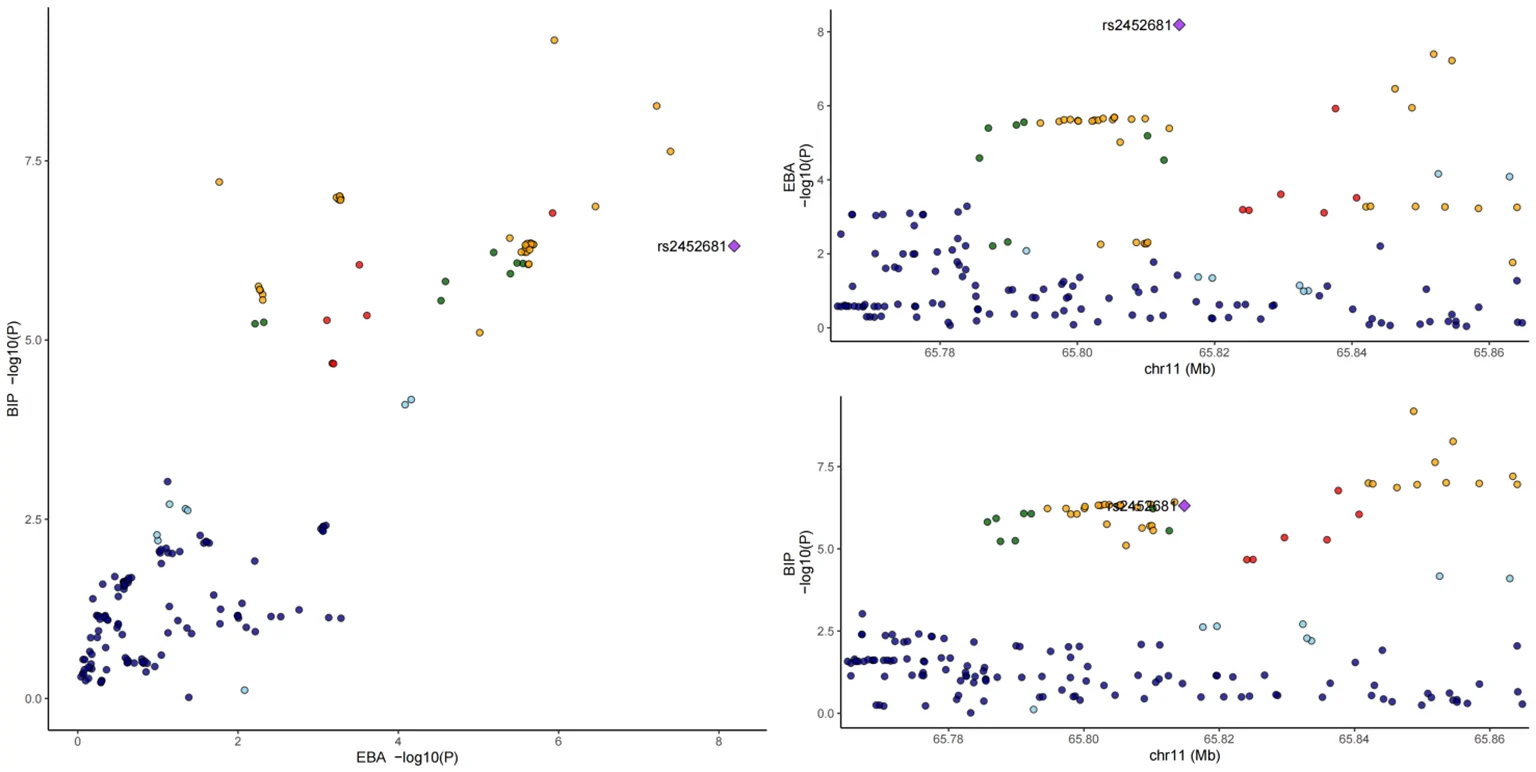
LocusCompare plot of the 11q13.1 locus between early pregnancy bleeding and bipolar disorder. Comparison of association signals at the 11q13.1 locus (lead SNP rs2452681) between EBA and BIP. The left panel shows a scatter plot of −log_10_(*P*) values for each SNP in both traits. The right panels show the regional association signals for EBA (top) and BIP (bottom) separately. SNPs are colored by r^2^ with the lead variant (purple diamond).

We next investigated cis-eQTL associations of the two novel loci using the GTEx v8 database to identify potential target genes and tissue-specific regulation (**Supplementary Table 9**). At the 15q15.1 locus (rs45457497)(**Figure 5**), the variant showed eQTL effects for multiple genes, including *CCDC32, DNAJC17, RAD51, RMDN3, RPAP1, RPUSD2, and ZFYVE19*. Among these, *CCDC32, RAD51*, and *RMDN3* exhibited significant expression in the brain, with *RMDN3* showing the broadest pattern across numerous regions—such as the hippocampus, caudate, anterior cingulate cortex (*BA24*), spinal cord (*cervical C-1*), substantia nigra, putamen, frontal cortex (*BA9*), cerebral cortex, amygdala, and nucleus accumbens. At the 11q13.1 locus (rs2452681) (**Figure 6**), eQTL analysis revealed multiple regulated genes, including *GAL3ST3, PACS1, SF3B2*, and *CTSF*. Several of these transcripts demonstrated significant expression effects in brain tissues, such as the frontal cortex, hippocampus, anterior cingulate cortex (*BA24*), putamen, and nucleus accumbens, as well as in the pituitary. Notably, *GAL3ST3* showed regulatory activity in both the pituitary and arterial tissues, whereas *PACS1* and *SF3B2* exhibited consistent expression modulation across multiple cortical and subcortical regions.

To further characterize the cellular context of these regulatory signals, we performed single-cell eQTL annotation using scQTLbase (9) (**Supplementary Table 10**). The lead variant rs45457497 at the 15q15.1 locus showed cell-type-specific regulatory effects involving *RMDN3* and nearby genes in immune-related cell populations, including natural killer cells and monocyte populations. The rs2452681 variant at the 11q13.1 locus was associated with cell-type-specific regulation of *PACS1*, *GAL3ST3*, and other nearby genes across immune cell populations, including B and T cells, as well as neurodevelopment-related cell populations such as ependymal-like cells and proliferating floor plate progenitors. These single-cell regulatory annotations provide additional cellular context for the prioritized loci and complement the tissue-level GTEx eQTL evidence.

Together, these findings suggest that the 11q13.1 locus may influence neurotransmission, neuroendocrine and synaptic pathways, potentially mediating the genetic overlap between psychiatric traits and reproductive outcomes.

## 3 Discussion

In this study, we conducted a comprehensive genetic investigation of early pregnancy bleeding by integrating genome-wide correlation, local genetic architecture, and multi-trait association analyses. By leveraging large-scale GWAS summary statistics across reproductive, metabolic, and psychiatric phenotypes, we demonstrate that early pregnancy bleeding has a complex and polygenic genetic basis that extends beyond reproductive biology. The significant genetic correlations with endometriosis, miscarriage, and uterine fibroids reinforce the roles of hormonal regulation and uterine tissue remodeling, whereas the strong positive correlations with psychiatric traits such as depression, ADHD, and PTSD highlight an emerging neuroendocrine dimension of early gestational complications.

Local genetic correlation analysis further refined these relationships and revealed several pleiotropic regions underlying early pregnancy bleeding and psychiatric traits. A prominent locus at 8q21.3 (*RUNX1T1*) showed convergent correlations across multiple psychiatric disorders, supporting a shared neurodevelopmental mechanism that links neuronal regulation to pregnancy maintenance (10). In addition, a strong correlation at 9p21 (*CDKN2A/B/ANRIL*)—a canonical vascular locus—suggests that genetic variation related to vascular integrity and endothelial remodeling may also contribute to the pathogenesis of early pregnancy bleeding (11).

Building on these insights, our multi-trait GWAS (MTAG) identified two novel loci—15q15.1 (rs45457497) and 11q13.1 (rs2452681)—that have not previously been implicated in either psychiatric or reproductive phenotypes. Of these, the 15q15.1 locus represents the most biologically compelling signal. The lead variant rs45457497 is a cis-eQTL for multiple genes, including *CCDC32, RAD51*, and particularly *RMDN3* (Regulator of Microtubule Dynamics 3), which shows broad expression across the brain, notably in the hippocampus, anterior cingulate cortex (*BA24*), amygdala, putamen, and frontal cortex. This pattern suggests that RMDN3 may represent a promising candidate gene potentially linking neuroendocrine regulation, stress responsiveness, and reproductive homeostasis. *RMDN3* (also known as *PTPIP51*) encodes a mitochondrial outer membrane protein that regulates ER–mitochondria tethering through interaction with *VAPB*, thereby modulating calcium homeostasis and autophagy (12). It is broadly expressed across brain regions, including the hippocampus, amygdala, and frontal cortex. Recent studies suggest that *RMDN3* plays a critical role in cellular stress responses, with enhanced ER–mitochondria coupling promoting neuronal resilience under stress, while dysregulation may contribute to aberrant calcium signaling and impaired autophagy (13, 14). These findings highlight RMDN3 as a prioritized candidate gene and suggest its potential involvement in the biological connection between neuroendocrine stress pathways and reproductive vulnerability.

Although sex-stratified LDSC analyses provided insights into potential differences in the magnitude of genetic overlap across male and female GWAS datasets, these findings should be interpreted with caution. Early pregnancy bleeding is a female-specific reproductive phenotype, and genetic correlations involving male-stratified traits do not imply that male-specific biological factors directly influence pregnancy outcomes. Instead, these associations likely reflect shared autosomal genetic architecture, whereby common genetic variants may contribute to both early pregnancy bleeding susceptibility and corresponding behavioral, psychiatric-related, or cardiometabolic traits. Differences in sample size, statistical power, and genetic architecture between male and female GWAS datasets may also contribute to the observed patterns, and further studies are needed to clarify the biological basis of these sex-dependent genetic correlations.

Taken together, our findings position early pregnancy bleeding as a multifactorial trait at the intersection of reproductive, neuroendocrine, and stress-related biology. The identification of *RMDN3* as a genetically and functionally prioritized candidate at a novel risk locus opens new avenues for understanding how brain–body signaling pathways influence maternal health outcomes and highlights the potential relevance of mental health-related genetic susceptibility and stress physiology in reproductive genetics research. More broadly, the identified genetic signals may reflect pleiotropic pathways connecting early pregnancy bleeding with psychiatric susceptibility, metabolic regulation, vascular function, and reproductive biology, rather than direct effects on early pregnancy bleeding alone. These findings provide potential genetic candidates for future studies integrating reproductive and psychiatric genetic susceptibility, which may help refine risk stratification approaches for pregnancy-related complications and mental health vulnerability.

## 4 Methods

### 4.1 GWAS data

Summary statistics for early pregnancy bleeding were obtained from a genome-wide association meta-analysis, comprising two phenotypes: Early Bleeding with Any outcome (EBA) and Early Bleeding ending in Live birth (EBL). Additional datasets included reproductive traits (endometriosis, uterine fibroids, and miscarriage), psychiatric disorders (ADHD, anxiety disorder, ASD, bipolar disorder, MDD, PTSD, and schizophrenia), and sex-stratified behavioral, metabolic, and anthropometric traits from the UK Biobank Neale Lab round 2 resource. Full details are provided in **Supplementary Table 1**.

### 4.2 Genetic correlation analysis

Genetic correlations (r_g_) between early pregnancy bleeding and reproductive traits, psychiatric traits, as well as metabolic, cardiovascular, and anthropometric traits were estimated using linkage disequilibrium score regression with corresponding GWAS summary statistics (15). Following standard analytical protocols, the analysis was restricted to HapMap3 SNPs, and precomputed linkage disequilibrium scores specific to these HapMap3 variants—derived from European-ancestry individuals in the 1000 Genomes Project—were employed to support the correlation estimates. LDSC, which calculates the r_g_ of additive genetic effects between two traits (with the sign indicating whether shared genetic effects act in the same or opposite directions on both traits), was also used to derive the SNP-based heritability of all examined traits. To ensure high data quality, SNP markers with an imputation INFO score below 0.9 were excluded prior to the analysis; additionally, it is noteworthy that a non-significant global genetic correlation from LDSC should not be interpreted as a lack of shared SNP associations between traits, as associations may still exist at the local or variant level. Multiple testing correction was performed using the Benjamini–Hochberg false discovery rate (FDR) procedure across all LDSC trait-pair analyses (n = 140), and both nominal P values and FDR-adjusted q values were reported.

### 4.3 Local genetic correlation analysis

To investigate local genetic correlations, we performed analyses using LAVA (16). Our analysis utilized the predefined segmentation of the genome into approximately independent 1 Mb loci provided by the LAVA framework. This local approach enables the identification of genetic correlations that are not detectable in genome-wide analyses, thereby providing a more granular perspective on complex genetic relationships. The analysis proceeded in two stages. First, univariate tests were conducted to estimate the local SNP-based heritability (h^2^SNP) for each trait. Only loci showing a significant local genetic signal for both traits were retained for subsequent analysis to ensure robust findings. Second, bivariate analyses were performed on these qualifying loci to compute the local genetic correlation between the trait pairs. The significance of bivariate correlations was assessed using the Benjamini–Hochberg false discovery rate (FDR) procedure (q < 0.05), applied separately within each trait category (reproductive, psychiatric, and cardiometabolic traits) based on the number of bivariate tests performed in each category.

### 4.4 Multi-trait GWAS analysis

MTAG was utilized to explore genetic associations between early pregnancy bleeding and psychiatric traits via a generalized meta-analysis framework, which boosts the power of detecting shared genetic loci by leveraging inherent genetic correlations among traits (3). The first step of the MTAG analysis involved rigorous variant filtering, where non-common SNPs, duplicated SNPs, and those with strand ambiguity were excluded to ensure the quality of genetic variants. After quality control and allele harmonization, only SNPs shared between the contributing GWAS datasets were retained for MTAG analysis. The numbers of input SNPs, post-QC SNPs, harmonized overlapping SNPs, and overlap proportions for each MTAG analysis are summarized in **Supplementary Table 11**. Next, pairwise genetic correlations between early pregnancy bleeding (designated as the primary trait) and psychiatric traits (treated as secondary traits) were estimated using LDSC, and these correlation estimates were further employed to calibrate the variance-covariance matrix of the random-effect component in the model. Following the matrix calibration, MTAG conducted a random-effect meta-analysis to generate updated SNP-level summary statistics. For identifying significant loci, SNPs were prioritized if they reached genome-wide significance (P < 5×10^-8^) in the multi-trait MTAG analysis and suggestive significance in the respective single-trait GWAS. Additionally, considering the complex linkage disequilibrium structure of the MHC region, it was treated as a single locus in the subsequent analyses to avoid potential biases from intricate genetic correlations within this region.

## Data Availability

The original contributions presented in the study are included in the article/**Supplementary Material**. Further inquiries can be directed to the corresponding author.
